# Assessing Gestation and Fetal Sex in Wild Assamese Macaques Using Urinary Estrogen Analysis

**DOI:** 10.1002/ajp.70065

**Published:** 2025-08-20

**Authors:** Verena Behringer, Suchinda Malaivijitnond, Suthirote Meesawat, Ruth Sonnweber, Michael Heistermann, Oliver Schülke, Julia Ostner

**Affiliations:** ^1^ Endocrinology Laboratory, German Primate Center Leibniz Institute for Primate Research Göttingen Germany; ^2^ Leibniz ScienceCampus Primate Cognition, German Primate Center Leibniz Institute for Primate Research Göttingen Germany; ^3^ Department of Biology, Faculty of Science Chulalongkorn University Bangkok Thailand; ^4^ National Primate Research Center of Thailand Chulalongkorn University Saraburi Thailand; ^5^ Vorarlberg State Institute for Environment and Food Safety Bregenz Austria; ^6^ Behavioral Ecology Department University of Goettingen Göttingen Germany; ^7^ Primate Social Evolution Group, German Primate Center Leibniz Institute for Primate Research Göttingen Germany

**Keywords:** estrone conjugates (E1C), fetal sex, field endocrinology, noninvasive hormone monitoring, reproductive monitoring

## Abstract

In mammals, estrogens and progestogens are crucial for gestation, fetal development, and maternal preparation for parturition and lactation. Measuring these hormones allows for the diagnosis of pregnancy, estimation of pregnancy failures, and potentially prenatal sex determination. We evaluated urinary estrogen and progestogen metabolites as biomarkers for gestation detection and for their utility for fetal sex determination in wild Assamese macaques (*Macaca assamensis*) using 586 samples from 19 females, including 19 successful pregnancies. Four enzyme‐immunoassays were tested for suitability in measuring urinary sex steroids using serial dilution: three assays targeting progestogen and one targeting estrogen metabolites (estrone conjugates, E1C). We performed a biological validation by measuring urinary hormone metabolites in one female across pre‐, early‐, late‐, and post‐gestation. None of the progestogen measurements reflected gestational status, while E1C levels showed the expected increases during gestation. Next, we measured urinary E1C across gestation in all females and investigated fetal sex effects on maternal E1C levels, expecting differences between females carrying male versus female fetuses. Urinary E1C levels increased as early as 9 days postconception and declined sharply at parturition, mirroring patterns in other primates. During late gestation, females carrying male fetuses had significantly higher E1C levels than those carrying female fetuses, yet overlapping values limit precision for prenatal sex determination. Urinary E1C offers a noninvasive marker for gestation monitoring in Assamese macaques, with application in ecological and conservation research. Additionally, results indicate intra‐ and inter‐species‐specific differences in steroid hormone metabolism and excretion, which need to be considered when selecting markers for reproductive monitoring.

## Summary

- Urinary estrone conjugates (E1C) are a reliable noninvasive biomarker for detecting gestation in wild Assamese macaques.
- E1C levels increase early in pregnancy (as soon as 9 days postconception) and drop sharply at parturition, reflecting patterns observed in other primates.
- Progestogen metabolite assays (PdG, 20α‐DHP, and pregnanolone) did not reliably indicate gestation status, highlighting species‐specific hormone excretion patterns.
- Females carrying male fetuses showed significantly higher E1C levels during late gestation, suggesting a potential link to fetal testicular activity.
- Substantial overlap in E1C values between sexes limits the predictive accuracy of prenatal fetal sex determination based on maternal urinary E1C.
- Steroid hormone excretion in Assamese macaques appears to be matrix‐specific, with estrogens excreted mainly in urine and progestogens likely in feces.
- Species‐specific hormone metabolism must be considered when selecting endocrine markers for reproductive monitoring in ecological and conservation research.

## Graphical abstract

Postable Summary: Urinary estrogen marks pregnancy in wild macaques, with limited use for sexing fetuses.

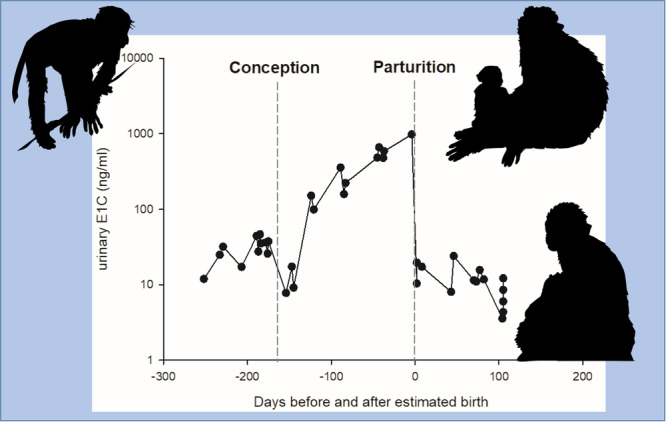

## 1 Introduction

The diversity and flexibility of nonhuman primate (hereafter “primate”) life‐history traits—such as age at first birth, gestation length, interbirth intervals, and weaning age — reflect the range of reproductive strategies shaped by the ecological and social environment. When considered alongside reproductive parameters like the number of ovarian cycles to conception, timing of ovarian activity, and cycle characteristics, these traits provide insights into primate evolution, ecology, and social behavior (Kamilar and Cooper 2013; Kappeler et al. 2003; Martin 2007). Reproductive parameters such as ovarian cycle characteristics, conception timing, and pregnancy progression can be assessed through hormone measurements. In particular, fluctuations in sex steroid hormones ‐ estrogens and progestogens ‐ are closely linked to key reproductive processes, including ovulation, implantation, and fetal development (Farage et al. 2009; Noyola‐Martínez et al. 2019; Saltzman et al. 2011). Tracking these hormonal patterns help monitoring cycle and/or pregnancy status and may also contribute to prenatal offspring sex determination (Higham 2016; Hodges and Heistermann 2011; Ostner and Heistermann 2003; Pethig et al. 2025).

Measuring hormonal fluctuations requires biological sample collection, which can be challenging, particularly in natural populations. While invasive techniques such as blood sampling provide direct hormone measurements, they are often impractical due to ethical concerns, stress‐related effects, and feasibility limitations. To overcome these challenges, noninvasive methods have become essential for monitoring reproductive states in both captive and wild populations. Noninvasive sampling minimizes stress and avoids behavioral disruptions. The potential stress response associated with invasive sample collection methods, such as drawing blood or plucking hair, as well as certain saliva collection techniques, could increase the risk of abortion or maternal death, especially in early pregnancies in some species of mammals (Peter et al. 2018). Feces and urine serve as key sample matrices for noninvasive reproductive monitoring, especially in large mammals such as primates (Behringer and Deschner 2017; Higham 2016). However, due to species‐specific differences in hormone metabolism and excretion, markers that reliably indicate reproductive status in one species or matrix may not necessarily perform similarly in others (Heistermann 2010; Hodges and Heistermann 2011; Whitten et al. 1998). To address these challenges, a diverse repertoire of hormonal markers is essential to build a comprehensive and adaptable toolbox for reliably monitoring reproductive states across species and contexts.

The sex steroid hormones, estrogens and progestogens, play pivotal roles in the regulation of female reproduction including pregnancy (Mesiano 2001). During primate gestation, the placenta interacts with maternal and fetal blood to produce estrogen estradiol and progesterone. Compared to the fluctuations observed across the ovarian cycle, estrogen and progesterone levels reach their highest concentrations during gestation (Farage et al. 2009; Levitz and Young 1978; Noyola‐Martínez et al. 2019). Estrogens prepare the uterus for implantation and contribute to placental development (Noyola‐Martínez et al. 2019; Saltzman et al. 2011). Progesterone supports implantation by modifying the endometrium, promoting the secretion of nutrients and growth factors that enhance embryo development. It also facilitates the formation of blood vessels crucial for placental attachment and nutrient exchange. In addition, progesterone modulates the maternal immune system by suppressing T‐cell activation, thereby reducing the risk of fetal rejection. It further contributes to pregnancy maintenance by inhibiting uterine contractions and suppressing lactation (Mesiano 2001). Therefore, progesterone is the main steroid for the maintenance of pregnancy.

In primates, monitoring sex steroid hormone metabolites such as pregnanediol‐3‐glucuronide (PdG) and/or estrone conjugates (E1C) in urine samples have been used for assessing ovarian function and reproductive states, including wild populations (Hodges and Heistermann 2011). In various primate taxa, including prosimians, platyrrhines, and catarhines, changes in urinary PdG levels have been applied to track ovarian cycles (e.g., Deschner et al. 2003; Emery Thompson 2005; Gerber et al. 2004; Savage et al. 1995; Shideler et al. 1990; Shimizu 2005), and to confirm pregnancies (e.g., Heistermann et al. 1996; Savage et al. 1995; Shimizu et al. 2003). Similarly, E1C, a major urinary metabolite of estradiol, has been widely used to monitor reproductive phases in various primate taxa (Heistermann 2010; Hodges et al. 2010). Monitoring changes in urinary E1C levels has proven effective for assessing ovarian activity, including the timing of ovulation (e.g., Deschner et al. 2003; Hashimoto et al. 2022; Karaskiewicz et al. 2023; Phillips and Wheaton 2008; Savage et al. 1995; Shimizu 2005), and to confirm and monitor pregnancy (e.g., Jarcho et al. 2012; Phillips and Wheaton 2008; Savage et al. 1995). While these markers have been successfully validated in many primate species, interspecific differences in hormone metabolism and excretion patterns highlight the need for species‐specific validation before applying them in reproductive monitoring.

Beyond their role in ovulation and pregnancy, assessment of sex steroids during gestation can inform us about the sex of the developing fetus, as their production is largely driven by the fetoplacental unit (Adamcová et al. 2018). Studies across primates have investigated how fetal sex influences maternal sex steroid hormone levels during pregnancy. For example, women carrying a female fetus had higher blood estradiol and 17‐OH‐pregnenolone levels compared to women carrying a male fetus (Adamcová et al. 2018; Toriola et al. 2011). In contrast, in lemur species, excreted maternal estrogen levels were higher during late gestation when carrying a male than a female fetus (Ostner and Heistermann 2003; Pethig et al. 2025; Shideler et al. 1983). The reasons for these contrasting patterns remain unclear, and potential mechanisms driving these associations across species require further investigation. While estrogen levels are associated with fetal sex, the direction of this effect appears to be species‐specific and warrants investigation in each primate species. Nevertheless, maternal estrogen levels have been found to be associated with fetal sex across different matrices and taxa.

While sex steroid analysis based on fecal samples has become a widely used noninvasive method in studies on macaque reproductive biology (e.g., Dubuc et al. 2009; Engelhardt et al. 2004; Fujita et al. 2001; Fürtbauer et al. 2010; Matsumuro et al. 1999), the potential of urinary hormone analysis remains underexplored. Urine represents an alternative noninvasive sample type that allows for frequent and standardized collection, making it particularly useful for endocrine monitoring in wild populations (Behringer and Deschner 2017; Higham 2016). To address this gap, we aimed to establish a noninvasive method to assess female reproductive status in wild Assamese macaques (*Macaca assamensis*) using urinary hormone analyses. Assamese macaques, like all macaque species, live in multimale‐multifemale groups with females remaining in their natal group throughout their lives and males dispersing (Anzà et al. 2022). Females of the study population at Phu Khieo Wildlife Sanctuary, Thailand, usually conceive with 5.5 years of age and are considered adult from this point on (Anzà et al. 2022; Fürtbauer et al. 2010; Touitou et al. 2021a). Reproduction follows a pronounced seasonal pattern with nearly 80% of births taking place between April and July (Fürtbauer et al. 2010; Touitou et al. 2021a). Gestation lasts on average 164 days, and interbirth intervals show a bimodal distribution with either ~14 or ~23 months between consecutive births (Fürtbauer et al. 2010; Shivani et al. 2025). Ovarian cycling ceases outside the mating season, and conceptions typically occur within a female's first or second ovulatory cycle (Fürtbauer et al. 2010). Although most females exhibit subcaudal sexual swellings, these do not reliably indicate reproductive status or fertility (Fürtbauer et al. 2010). Lactation lasts the entire first year of an infant's life, but time spent in nipple contact during the day already strongly decreases during the first 6 months (Arbaiza‐Bayona et al. 2025; Berghänel et al. 2016).

The validation and establishment of urinary biomarkers for pregnancy and prenatal sex determination could provide a valuable tool for estimating abortion rates, assessing fitness consequences for females, and evaluating prenatal sex ratios. To explore this potential, we analyzed 586 urine samples collected from 19 wild female Assamese macaques over the course of a reproductive year. First, we biochemically validated four enzyme immunoassays using serial dilution of urine samples: three progestogen assays (20α‐dihydroprogesterone [20alpha‐OHP], PdG, and pregnanolone) and an estrogen assay for E1C. Second, we conducted a biological validation by comparing samples from pre‐, early‐, late‐ and post‐gestation of a single female for three of the assays, while for the fourth assay (pregnanolone), samples of a different female were used. We expected increased levels of both progestogens and estrogens during pregnancy compared to the other reproductive states. Third, we used the E1C assay to monitor estrogen changes across 19 successful pregnancies, assessing both the timing and magnitude of gestational E1C increases to determine whether a fixed threshold could reliably indicate a pregnant status. Finally, we tested whether we could reliably determine fetal sex prenatally based on E1C measurement during late gestation.

## 2 Materials and Methods

### Study Population and Sample Collection

This study is part of a long‐term research project on a population of Assamese macaques at Phu Khieo Wildlife Sanctuary (16° 5′–35′ N, 101° 20′–55′ E), in northeastern Thailand, part of the > 6500 km^2^ Western Isaan Forest Complex, a connected and protected forest area. Phu Khieo Wildlife Sanctuary holds the highest protection status under Thailand conservation regulation for flora and fauna. The local study site, Huai Mai Sot Yai (16° 27′ N, 101° 38′ E), is characterized by hilly terrain at elevations of 600–800 m a.s.l. and consists primarily of hill and dry evergreen forest, interspersed with patches of bamboo and dry dipterocarp forest (Borries et al. 2002; Koenig et al. 2004; Kumsuk et al. 1999).

The climate at the study side is marked by a cold, dry season from November to mid‐March, with minimum temperatures occasionally approaching freezing in January (lean season). This is followed by a warm, rainy season, which receives an average annual precipitation of 1374 mm/year, peaking in May and September (rich season) (Richter et al. 2016). Although fruit and food availability increase during the rich season, resource availability fluctuates annually, and forest productivity is considered highly unpredictable (Berghänel et al. 2016; Heesen et al. 2013; Schülke et al. 2011; Touitou et al. 2021a). The study population is primarily frugivorous, with the majority of their plant diet consisting of fruit, pulp, and seeds. A considerable portion of their feeding budget is dedicated to slow and low‐intensity foraging for animal matter (Heesen et al. 2013; Schülke et al. 2011; Touitou et al. 2021b).

A total of 586 urine samples were collected between June 2017 and January 2019 from 19 adult females living in three social groups (median = 32; range=19‐41; mean = 30.8; standard deviation (SD) = 6.5; Supplementary Table S1). Samples were collected opportunistically throughout the day using disposable mini‐pipettes. To avoid collection in low light conditions, the first‐morning void was never collected, as it was too dark to reliably identify the individuals when they were high in the canopy. Only urine free from fecal contamination was sampled. The samples were pipetted either from clean, disposable plastic bags placed underneath the females during urination or directly from vegetation. The urine was then transferred into 2 mL Eppendorf vials, which were labeled with the date, time of collection, and female ID (Touitou et al. 2021b). Specific gravity (SG) was measured immediately after collection (Anestis et al. 2009; Miller et al. 2004), with a few drops of urine placed on the lens of a handheld refractometer (Atago PAL–10S), and after the SG measurement, the urine was returned to the vial, which was sealed with Parafilm. The lens was cleaned with Kimtech wipes between measurements. To maintain sample integrity, the urine was kept cool in the forest using Thermos flasks filled with ice cubes. At the end of each day, the samples were stored in a freezer at the camp ( − 12°C) before being transported to a second freezer ( − 19°C) in a nearby village (Touitou et al. 2021b). Samples were kept frozen until being shipped to the endocrinology laboratory of the German Primate Center, Göttingen, Germany, for hormone analysis.

### Hormone Measurement

We validated four in‐house enzyme immunoassays (EIAs) for the quantification of reproductive hormone metabolites in urine samples of our study species: three progestogen assays measuring 20α‐OHP, PdG, and pregnanolone, as well as an estrogen assay for E1C. Hormonal analyses were conducted using un‐extracted urine. Urine samples were thawed and diluted according to assay‐specific requirements. Samples analyzed for 20α‐OHP, PdG, and pregnanolone were diluted 1:80 with 0.04 M phosphate buffered saline, while those measured for E1C underwent dilution steps ranging from 1:50 to 1:3200, depending on concentration. For the serial dilution tests, samples were diluted 1:80, 1:160, 1:320, 1:640, 1:1280, and 1:2560 for each assay, respectively.

The 20α‐OHP assay has been described in detail in Heistermann et al. (1995) and used for our study population previously using fecal extracts (Fürtbauer et al. 2010). The antiserum for the measurement of 20α‐OHP was raised in a rabbit against 20α‐OHP‐20‐CMO‐BSA. Biotinylated 20aOHP in conjunction with peroxidase labelled streptavidin was used as conjugate. Pregnanolone assay procedure and performance characteristics have been described previously (Fürtbauer et al. 2020; Graham et al. 2001; Munro and Stabenfeldt 1984). The pregnanolone assay uses the monoclonal antibody CL425 (final purification by C. Munro, Davis, CA) and is considered a promising tool for monitoring progesterone and its metabolites in urine and feces across a range of species (e.g., Amendolagine et al. 2018; Ditcham et al. 2009; summarized in Graham et al. 2001). Assay procedure and performance characteristics have been described previously (Graham et al. 2001; Munro and Stabenfeldt 1984). E1C and PdG were described in detail in Heistermann and Hodges (1995). Assay characteristics, including antibody cross‐reactivities and sensitivities, are described in Heistermann et al. (1995) and in Heistermann and Hodges (1995). The EIA method for measuring urinary E1C has been validated in multiple species, showing parallel changes with plasma estradiol (Munro et al. 1991; Shideler et al. 1990; Shimizu 2005). The E1C assay utilizes an antiserum raised in a rabbit against estrone‐3‐glucuronide‐BSA, with sheep anti‐rabbit IgG (No. R‐9754 Sigma Chemie) as the coating antibody and estrone‐3glucuronide labelled with alkaline phosphatase as the conjugate. The PdG assay uses an antiserum raised in a rabbit against pregnanediol‐3‐glucuronide‐BSA and biotinylated PdG as the label.

All samples were run in duplicates, CV between duplicates never reached more than 9%, thus no duplicate measurements were excluded from the analysis. Inter‐assay and intra‐assay coefficients of variation determined from quality controls and samples, respectively, are provided in Table 1. All measurements were corrected for SG to adjust for urinary concentration (Miller et al. 2004). Results are reported as corrected (corr.) concentrations in ng/ml corr. SG. Only samples with a SG > 1.003 were included in the analysis to exclude too diluted samples.

**Table 1:** Inter‐assay and intra‐assay coefficients of variation (CV) determined from quality controls and samples, respectively, for the four enzyme immuno assays. 20α‐dihydroprogesterone = 20alpha‐OHP, pregnanediol‐3‐glucronide = PdG, estrone conjugates = E1C.

Inter‐assay				Intra‐assay	
	CV high	CV low	No. plates	CV	No. samples
20alpha‐OHP	5.9%	4.1%	2	3.0%	27
PdG	2.0%	0.4%	2	2.0%	26
Pregnanolone	8.1%	13.8%	3	2.5%	35
E1C	4.9%	9.1%	20	2.2%	35

### Statistical Analysis

#### 2.1 Data Preparation

The estimated day of conception was calculated as the day of parturition minus 164 days, the average gestation length for this study population (Fürtbauer et al. 2010). Gestation was divided into two phases: early gestation (first 82 days after conception) and late gestation (82 days before parturition) (Touitou et al. 2021b). We averaged urinary E1C measurements for each female for each of the four reproductive stages: pre‐gestation (pre), early gestation, late gestation, and post‐gestation (post).

#### 2.2 E1C Levels Across a Reproductive Year

To compare E1C levels across reproductive stages, we first assessed data distribution using the Shapiro‐Wilk test for normality. As E1C values were not normally distributed, we conducted a Kruskal‐Wallis one‐way analysis of variance (ANOVA) on ranks. Post‐hoc pairwise comparisons were performed using a Dunn's Method without adjustment for ties.

#### 2.3 Calculating a Threshold for Pregnancy Determination

To determine a potential E1C threshold for differentiating between a nonpregnant and pregnant status, the mean + 2 standard deviations (SDs) was calculated using all pre‐pregnancy samples collected in November and December 2017, the 2 months during which nearly all conceptions occurred. Sample values exceeding this defined non‐pregnancy threshold are most likely indicative of pregnancy, given that > 95% of data points in a population lie within 2 SDs of the mean (Upton and Cook 2008).

#### 2.4 Assessing the Effect of Fetal Sex

To assess the effect of fetal sex on E1C levels, we conducted separate two‐tailed *t*‐tests for each reproductive stage (pre‐, early‐, late‐, and post‐gestation), comparing females carrying male versus female fetuses. Normality was tested for each comparison, and *t*‐tests were applied when assumptions were met.

For all statistical analyses, a *p* value of < 0.05 was used as the criterion for statistical significance.

## 3 Results

### Serial Dilution

**Figure 1:**
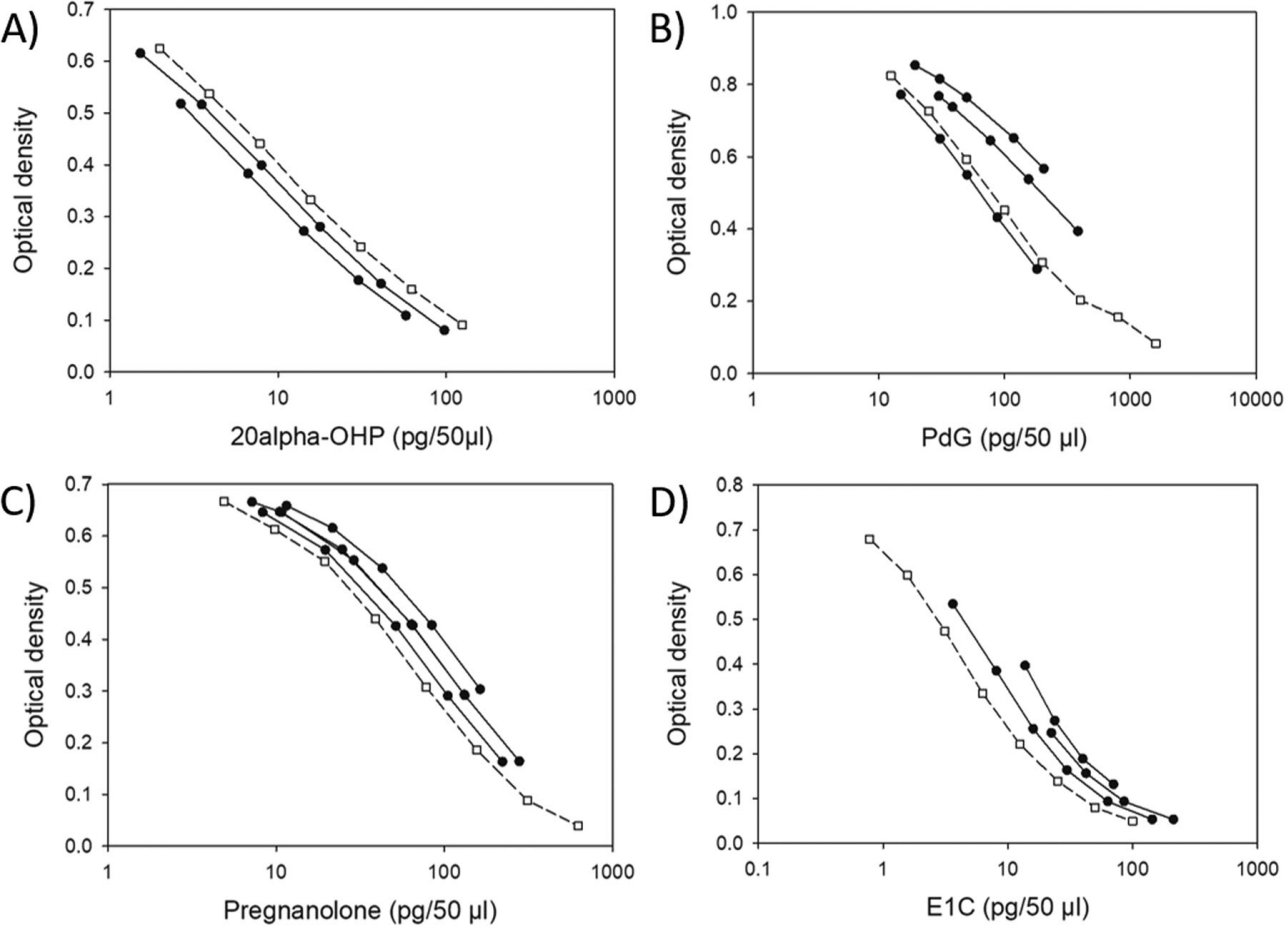
Concentrations of standards and serial dilutions of samples (pg/50 µL). Linearity of four steroid metabolites (A) 20α‐dihydroprogesterone (20α‐OHP), (B) pregnanediol‐3‐glucronide (PdG), (C) pregnanolone, and (D) estrone conjugates (E1C). Standard curves (dotted lines, open squares) and serial diluted urine samples (straight lines, filled circles).

Serial dilutions of urine samples from the pre‐gestation, gestation, and post‐gestation period were analyzed using the 20α‐OHP, PdG, pregnanolone, and E1C assays. The resulting displacement curves paralleled the respective standard curves in all assays, with the exception of a slight deviation in the PdG assay, suggesting minimal interference from the urinary matrix (Figure 1).

### Biological Validation

**Figure 2:**
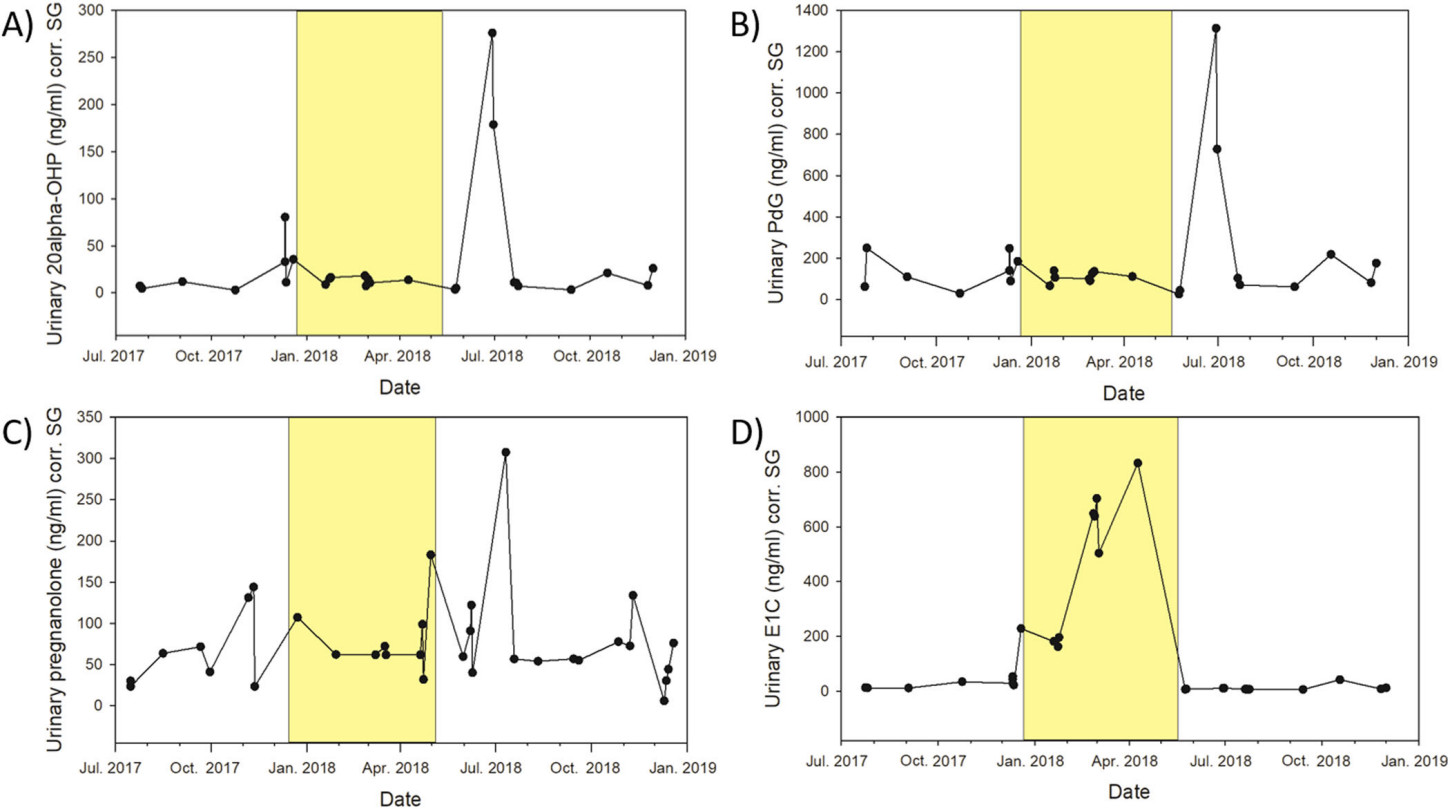
Urinary metabolites profiles of two females over the course of more than a year (from July 2017 to December 2018), measured with a (A) 20α‐dihydroprogesterone (20α‐OHP), (B) pregnanediol‐3‐glucronide (PdG), (C) pregnanolone, and (D) estrone conjugates (E1C) assay. The yellow background highlights the period of gestation.

For biological validation, we measured hormone levels in urine samples collected from two females over a 1‐year period, including pregnancy. For this step, we selected the two females who, on the one hand, had the highest number of urine samples across the 1‐year period and, on the other hand, provided sufficient urine volume per sample to allow for repeated measurements using the different assays. We expected higher urinary estrogen and progesterone metabolite levels during pregnancy compared to nonpregnant phases. While E1C levels increased during pregnancy as anticipated, none of the progestogen measurements showed the expected increase (examples provided in Figure 2). In all three progestogen profiles, an unexpected peak occurred after parturition. We can only speculate about its causes, but two potential explanations seem unlikely. First, an adrenal contribution to urinary progestogen levels is unlikely as hormone concentrations were low in first post‐parturition samples. Second, early resumption of ovarian cycling seems biologically implausible as Assamese macaques are seasonal breeders and postpartum ovulation outside the mating season has not been reported. Alternatively, the observed peak could reflect assay cross‐reactivity with other postpartum steroids or shifts in hormone metabolism and excretion that affect urinary metabolite levels independently of circulating hormone concentrations. As only the E1C assay provided the biologically expected pattern, for the following analyses we focused on E1C levels in samples of the 19 study females.

### E1C Levels Across a Reproductive Year

Following estimated conception (164 days before parturition), urinary E1C levels were initially low in all 19 females (Figures supplement). The earliest increase in urinary E1C levels occurred after 9 days past conception in one female (female ID: PAI, Figure supplement), however, on average, E1C levels started to increase about 3 weeks after conception (Figures in the supplement). Overall, E1C levels increased on average 21‐fold when pregnancy progresses, that is, from 25.2 ng/mL corr. SG (SD = 21.9 ng/mL) pre‐gestation to 532.6 ng/mL corr. SG (SD  =  289.3 ng/mL) in late‐gestation, reaching maximum levels shortly before parturition.

**Figure 3:**
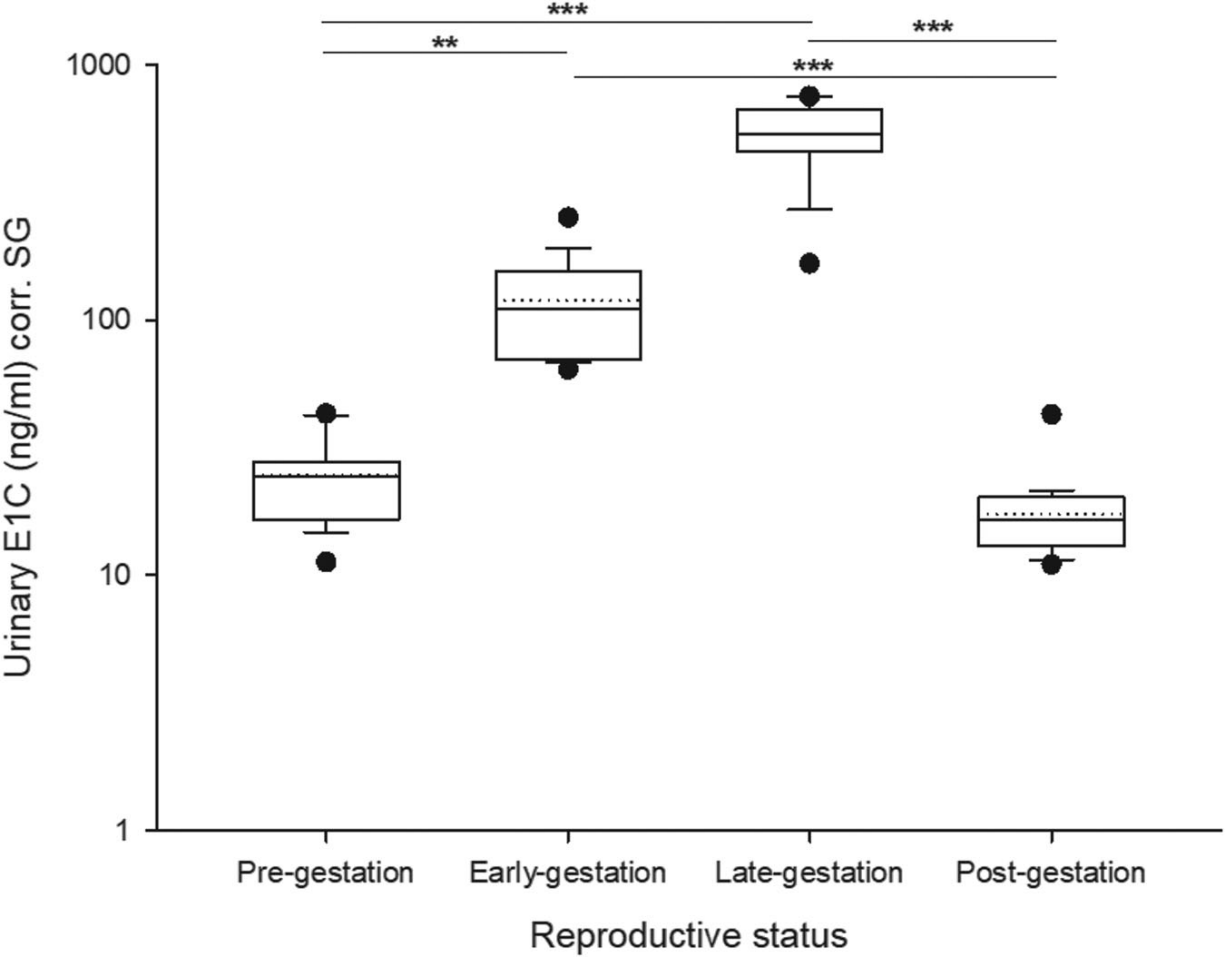
Distribution of urinary estrone conjugates (E1C) levels of 19 female Assamese macaques across reproductive stages (pre‐, early‐, late‐ and post‐gestation). The y‐axis is displayed on a log scale. Boxes illustrate the 25th and 75th percentiles, dashed lines indicate medians, solid lines indicate means, and circles present outliers. * = *p* < 0.05, ** = *p* < 0.01, *** = *p* < 0.001.

Reproductive state significantly predicted differences in median E1C levels (Kruskal‐Wallis one‐way ANOVA on Ranks, H = 65.1; df = 3; *p* = < 0.001). Post‐hoc pairwise comparisons revealed higher E1C levels during both early‐ and late‐gestation compared to pre‐ and post‐gestation (Figure 3, Table 2). No statistical differences were found between early and late gestation, or between pre‐ and post‐gestation stages (Table 2).

**Table 2:** Results of Kruskal‐Wallis one‐way ANOVA on ranks for urinary E1C levels (ng/mL urine) of 19 female Assamese macaques across reproductive stages (pre‐, early‐, late‐ and post‐gestation). Post‐hoc Dunn's Method for multiple pairwise comparison. Significant results are indicated in bold.

Reproductive state	Median	25%	75%
Pre‐gestation	24.4	16.5	28
Early‐gestation	110.5	70.5	155.5
Late‐gestation	536.9	457.9	669.9
Post‐gestation	16.6	13.0	20.3
**Comparison**	**Diff. of ranks**	**Q**	***p***
Late versus post	52.4	7.309	**< 0.001**
Late versus pre	42.4	5.921	**< 0.001**
Late versus early	18.8	2.622	0.052
Early versus post	33.6	4.687	**< 0.001**
Early versus pre	23.6	3.298	**0.006**
Pre versus post	9.9	1.388	0.990

The mean E1C concentration of all nonpregnant samples was 41.7 ± 27.4 ng/mL corr. SG. Accordingly, the mean + 2 SDs (used as thresholds to differentiate nonpregnant from pregnant states, see above) was 96.5 ng/mL corr. SG. This threshold was exceeded in approximately 40% of the samples collected during the first half of gestation and in 100% of the samples collected during the second half of pregnancy.

**Figure 4:**
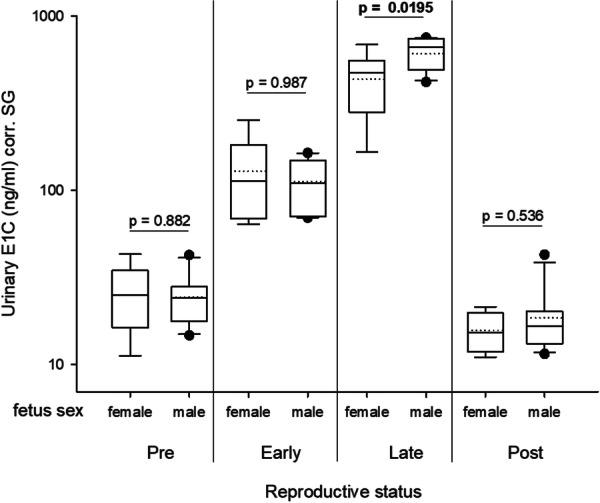
Urinary estrone conjugates (E1C) levels of female Assamese macaques carrying a female (*N* = 8) or a male (*N* = 11) fetus across reproductive stages (pre‐, early‐, late‐, or post‐gestation). The y‐axis is displayed on a log scale. The boxes illustrate the 25th and 75th percentiles, dashed line medians, solid lines means, and circles represent outliers.

We further compared average E1C levels in females carrying a male fetus (*N* = 11) versus a female fetus (*N* = 8) across reproductive stages (pre‐, early‐, late‐, post‐gestation). A two‐tailed t‐test showed that, despite overlapping values, females carrying a male fetus had significantly higher E1C levels during late gestation (male fetus = 608.61 ng/ml; female fetus = 435.14 ng/ml; *t* = −2.579; df = 17; *p*‐value = 0.0195; Figure 4). In all other reproductive stages, fetal sex had no significant effect on maternal urinary E1C levels (all *p* > 0.05, Figure 4).

## 4 Discussion

Our study identified urinary estrone conjugates (E1C) as a reliable noninvasive marker for tracking gestational hormonal changes in wild Assamese macaques, while urinary progestogen measurements did not reflect expected pregnancy‐related increases. Urinary E1C levels increased shortly after conception and remained elevated throughout gestation. Notably, higher late‐gestation E1C levels were observed in mothers carrying male fetuses, although substantial overlap between sexes limits reliable prenatal sex determination.

While urinary E1C proved to be a promising endocrine marker reflecting hormonal changes during gestation in wild‐living Assamese macaques, urinary progestogen measurements failed to detect the expected increase in progestogen levels during pregnancy. These findings contrasts with previous results measuring estrogen and progesterone metabolites in fecal samples of pregnant females of this species, which demonstrated elevations in progestogen, but not estrogen, levels during gestation (Fürtbauer et al. 2010). The reason for these contrasting findings remains less clear; however, the results suggest that in Assamese macaques, the excretion pathway of estrogen and progesterone metabolites may differ substantially, with estrogens predominantly excreted via urine and progestogens mainly via feces. The plausibility of such a scenario is supported by research on female cotton‐top tamarins (*Saguinus oedipus oedipus*), in which a radiometabolism study showed that approximately 90% of labeled estradiol is excreted into the urine, whereas 95% of labeled progesterone is excreted into the feces (Ziegler et al. 1989). Species‐specific excretion patterns are further supported by findings in squirrel monkeys (*Saimiri sciureus*), in which both progestogens and estrogens are mainly excreted in feces (Moorman et al. 2002), whereas in rhesus macaques (*M. mulatta*), progesterone (42% recovery in urine) and estradiol (55% recovery in urine) show nearly equal urinary and fecal excretion (Shideler et al. 1993). Those and our results, in combination with previous findings on Assamese macaques, indicate two key points: First, species‐specific differences influence hormone excretion patterns. Second, in our study species, estrogens appear to be primarily excreted in urine, while progestogens are likely excreted predominantly in feces. Altogether, these findings emphasize the importance of considering sample matrix when selecting hormonal markers for reproductive monitoring.

Our findings demonstrate that urinary E1C measurement is a valuable tool for monitoring endocrine changes associated with pregnancy in female Assamese macaques. Following estimated conception, urinary E1C levels were initially low before increasing around 2–3 weeks postconception. A similar pattern has been observed in female rhesus and Tonkean macaques (*M. tonkeana*), where estrogen levels in urine and serum declined after conception before increasing 16–18 days later (Monfort et al. 1987; Thierry et al. 1996). This early rise in urinary estrogen levels, typically occurring around 2–3 weeks postconception, has been documented across various primate species (Czekala et al. 1981, 1983; Hodgen et al. 1972), including humans (Ahmed and Kellie 1972; Mesiano 2001; Venners 2006). Estrogen levels show a pronounced increase during the second half of gestation, reaching their highest concentrations recorded in the weeks shortly before parturition. This marked elevation of estrogen levels in late gestation is a typical characteristic of primate pregnancies (e.g., Monfort et al. 1987; Shimizu and Mouri 2018; Thierry et al. 1996) and can largely be attributed to the endocrine activity of a functioning fetoplacental unit (Mesiano 2001). Therefore, urinary estrogen assessment throughout gestation in Assamese macaques may serve as an indicator of fetal health, as has been suggested for other primate species (Czekala et al. 1988; Thierry et al. 1996). While previous research has successfully validated fecal samples for assessing reproductive status (Fürtbauer et al. 2010), our study expands this approach by demonstrating that urinary E1C can serve as a reliable alternative broadening the toolkit available for noninvasive reproductive monitoring in this species.

The substantial increase in E1C levels during gestation compared to nonpregnant levels allows pregnancy diagnosis using only a few, or even a single, sample (Hidayatik et al. 2018; Scheun et al. 2016). Our data suggest that urinary E1C levels exceeding 100 ng/mL corr. SG indicate pregnancy with > 95% certainty. This cut‐off value is reliably exceeded from approximately the second half of gestation, suggesting that pregnancy diagnosis from a limited number of urine samples can be performed with high accuracy from about the third month of gestation onwards. This capability is valuable for estimating pregnancy rates in wild Assamese macaques and facilitates understanding of social and environmental factors affecting reproductive parameters, such as fetal loss and population reproductive health under natural conditions.

In the second half of gestation, urinary E1C levels were significantly higher in Assamese macaque mothers carrying male fetuses compared to those carrying female fetuses. This finding is consistent with studies in different species of lemurs, where elevated estrogen levels were observed in the last weeks of gestation in females carrying a male fetus. Specifically, increased estrogen excretion has been detected in females carrying male fetuses of red‐fronted lemurs (*Eulemur rufifrons*) (Ostner et al. 2003; Ostner and Heistermann 2003; Pethig et al. 2025), black‐and‐white ruffed lemurs (*Varecia variegata*) (Shideler et al. 1983), red‐bellied lemurs (*E. rubriventer*), blue‐eyed black lemurs (*E. macaco flavifrons*), and eastern lesser bamboo lemurs (*Hapalemur griseus*) (Gerber et al. 2004). Given that estrogens are derived from androgen precursors, it has been suggested that the increased estrogen levels observed during late pregnancy in mothers carrying a male fetus are presumably relate to the onset of testicular androgen production in those fetuses (Shideler et al. 1983). However, in our study, E1C levels in mothers carrying a male or a female fetus overlapped considerably, and unlike the distinct patterns previously reported in lemurs, this extensive overlap introduces substantial uncertainty, rendering prenatal sex determination in Assamese macaques unreliable.

While our study offers novel insights into gestational hormone patterns and the potential for prenatal sex assessment in wild Assamese macaques, several limitations should be acknowledged. First, the absence of daily sampling around conception and parturition likely limited the temporal precision of our hormonal profiles. Second, although our sample size is among the largest for wild primates in this context, it remains relatively small for evaluating subtle individual variation, particularly in fetal sex comparisons. Finally, our data represent a single wild population, and species‐ or population‐level variation in hormone metabolism and excretion may limit broader generalization. Nevertheless, our results provide a strong foundation for future work on the reproductive biology of wild‐living Assamese macaques.

## 5 Conclusion

Our study establishes urinary E1C as a noninvasive marker for monitoring gestation status in wild Assamese macaques. The observed estrogen excretion patterns throughout pregnancy align with those reported in other primates, supporting the reliability of urinary E1C measurement for endocrine diagnosis and monitoring of gestation. Notably, E1C levels exceeding 100 ng/mL corr. SG identified pregnancy with > 95% certainty from mid‐gestation onward, enabling reliable diagnosis from few urine samples from the third month of gestation. Additionally, our findings suggest potential intraspecific variation in hormone excretion pathways, with estrogens presumably excreted predominantly via urine, while progestogens are presumably eliminated primarily via feces. The elevated urinary E1C levels in mothers carrying male fetuses during late gestation suggest a potential influence of fetal testicular precursors on maternal estrogen levels. However, the overlap in E1C values between male‐ and female‐carrying mothers indicates that prenatal fetal sex determination based on urinary E1C carries considerable uncertainty in this species. From a physiological perspective, these results emphasize the importance of considering species‐specific hormone metabolism and excretion patterns when choosing a suitable endocrine marker for reproductive monitoring.

## Author Contributions

**Verena Behringer:** conceptualization, formal analysis, investigation, methodology, validation, visualization, resources, writing – original draft. **Suchinda Malaivijitnond:** project administration, writing – review and editing. **Suthirote Meesawat:** project administration, writing – review and editing. **Ruth Sonnweber:** formal analysis, writing – review and editing. **Michael Heistermann:** conceptualization, methodology, validation, writing – review and editing. **Oliver Schülke:** conceptualization, funding acquisition, methodology, project administration, resources, writing – review and editing. **Julia Ostner:** conceptualization, methodology, investigation, project administration, resources, funding acquisition, writing – review and editing.

## Ethics Statement

This study was conducted entirely non‐invasively and complied with the ASAB/ABS Guidelines for the Use of Animals in Research (https://www.asab.org/ethics). All research protocols were approved by the appropriate Thai authorities: the Department of National Parks, Wildlife and Plant Conservation and the National Research Council of Thailand, which also authorized data collection and the export of samples under a benefit‐sharing agreement (permit numbers: 0002/4137, 0402/2798). The research adhered to the American Society of Primatologists (ASP) Principles for the Ethical Treatment of Nonhuman Primates and followed the ASP Code of Best Practices for Field Primatology.

## Conflicts of Interest

The authors declare no conflicts of interest.

## Supporting information

Supplement Figures all 19 E1C profiles.

Supplement Table S1.

## Data Availability

The data that support the findings of this study are available from the corresponding author upon reasonable request.
