# Supplementary material for: Assessing Gestation and Fetal Sex in Wild Assamese Macaques Using Urinary Estrogen Analysis
Source: Am J Primatol. 2025 Aug 20;87(8):e70065. doi: 10.1002/ajp.70065 (PMC12367238; doi:10.1002/ajp.70065)
Supplement: Supplementary file 1 — Supplement Figures all 19 E1C profiles. [file AJP-87-e70065-s002.pdf]

E1C profiles of 19 Assamese macaque females

All y-axes are in urinary E1C ng/ml concentrations adjusted for specific gravity. C = conception (bright blue), HG = half gestation (bright green), P = parturition.

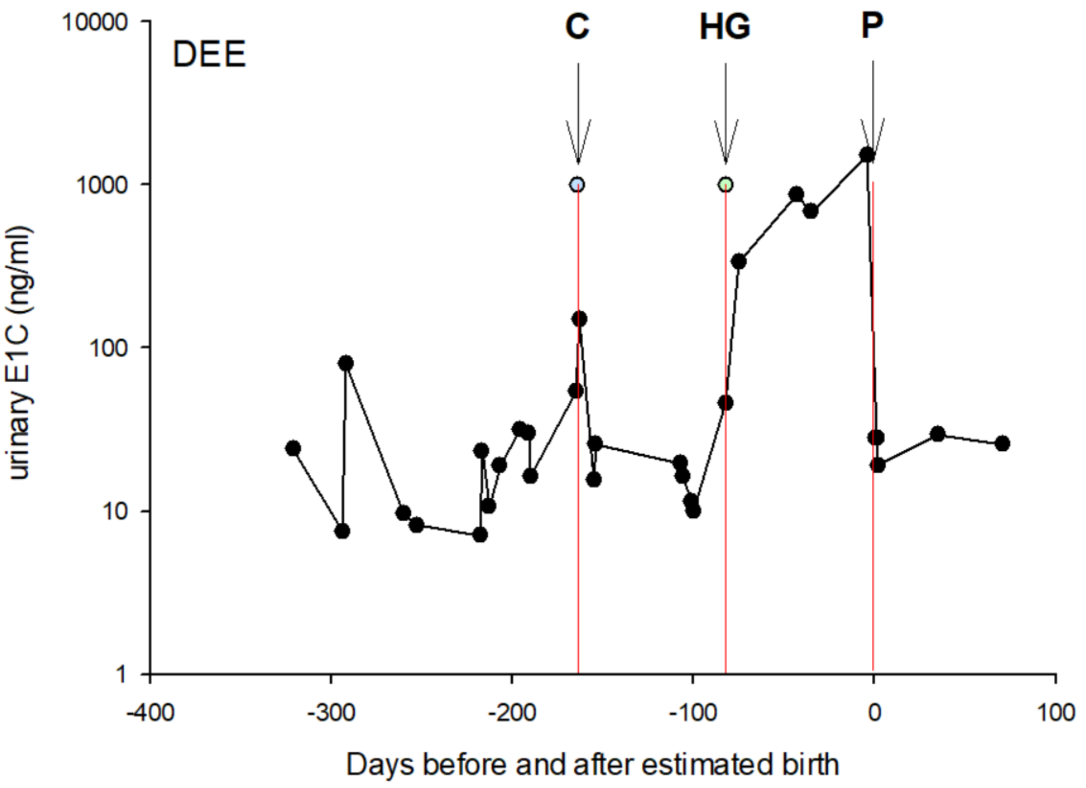

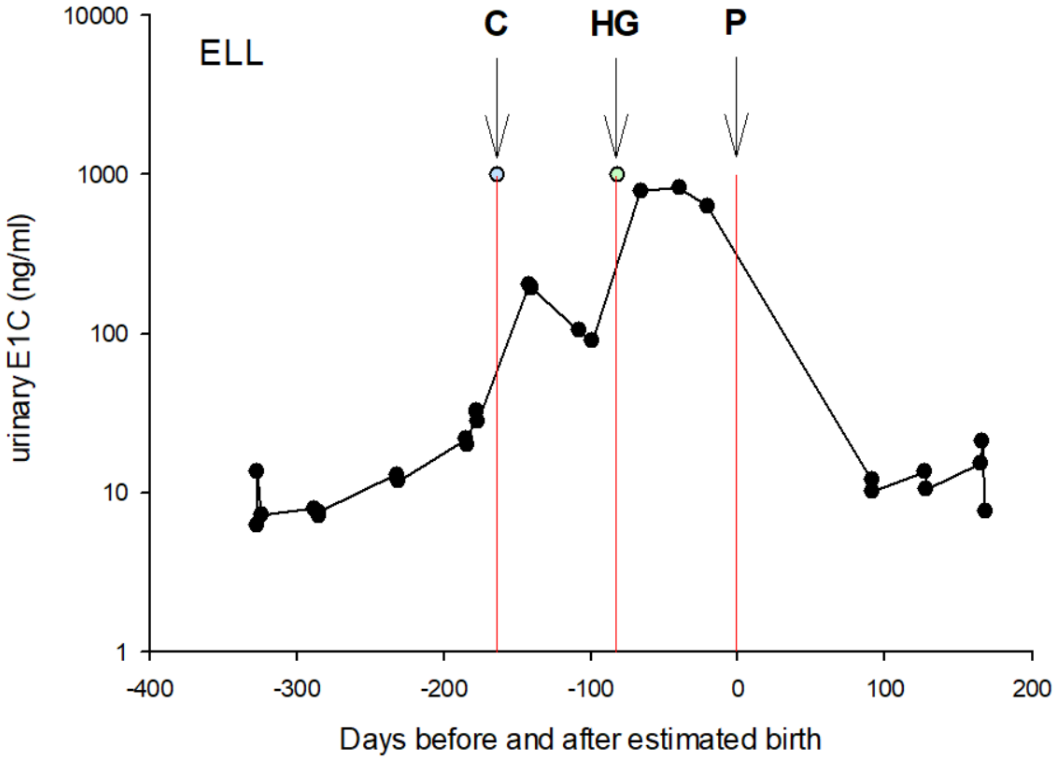

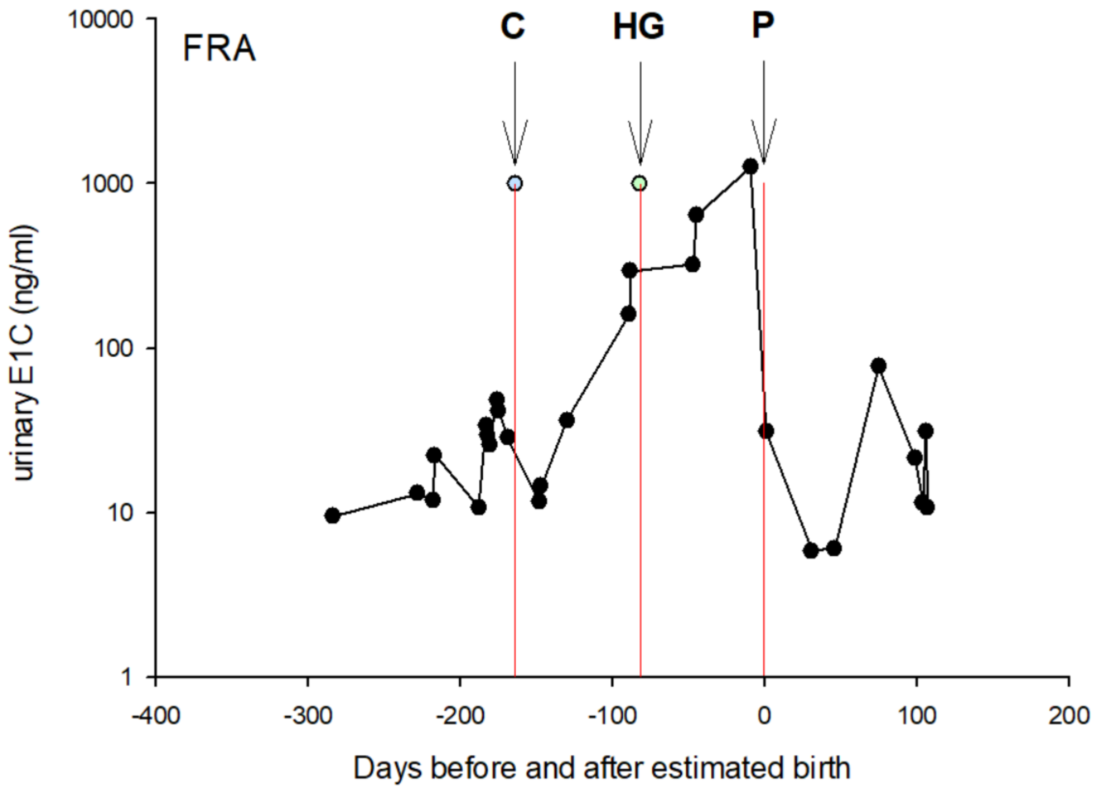

FRA
C
HG
P
10000
1000
100
10
1
urinary E1C (ng/ml)
-400
-300
-200
-100
0
100
200
Days before and after estimated birth

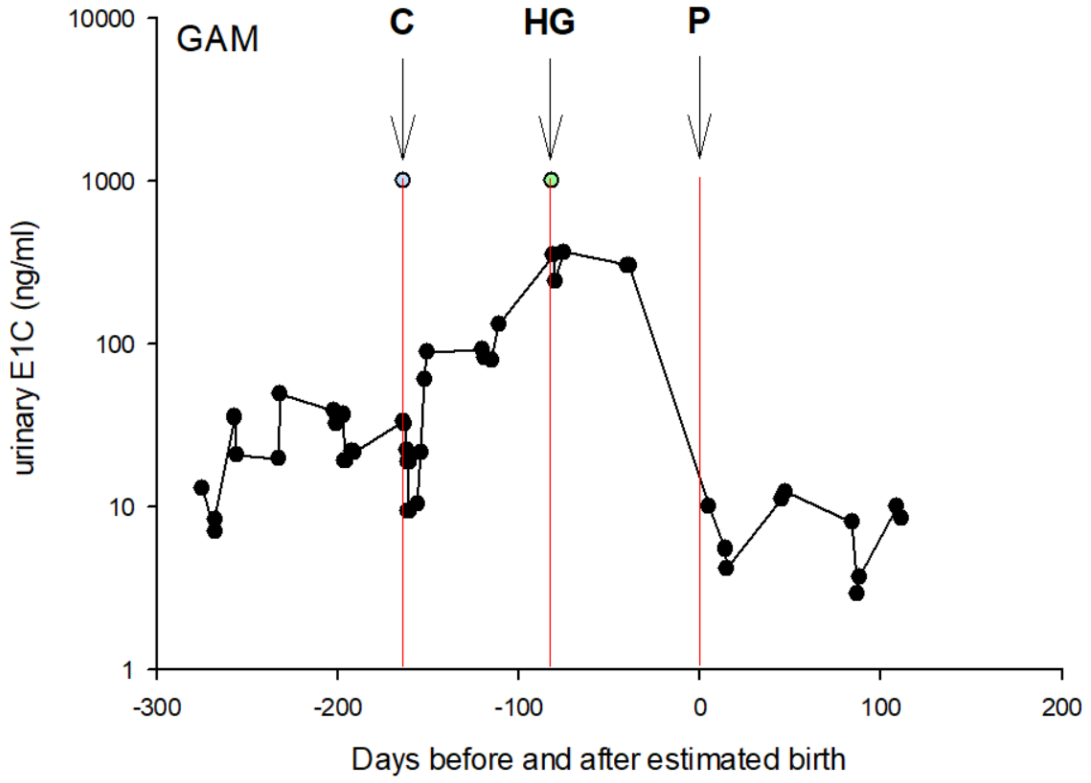

GAM
C
HG
P
10000
1000
100
10
1
urinary E1C (ng/ml)
-300
-200
-100
0
100
200
Days before and after estimated birth

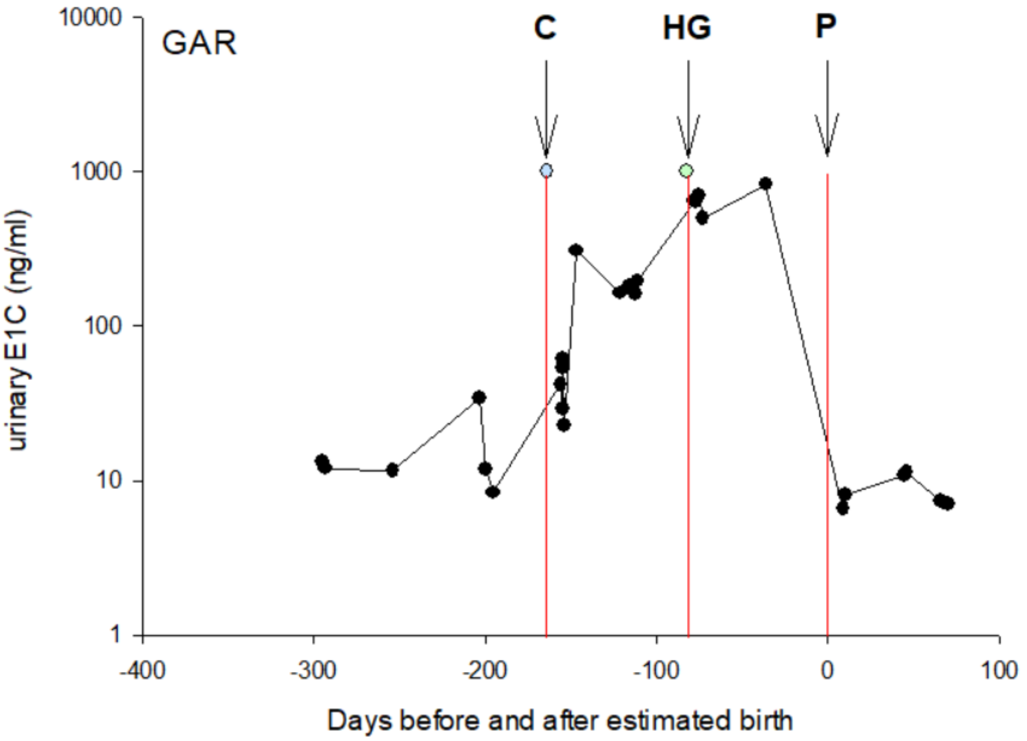

GAR
C
HG
P
10000
1000
100
10
1
urinary E1C (ng/ml)
-400
-300
-200
-100
0
100
Days before and after estimated birth

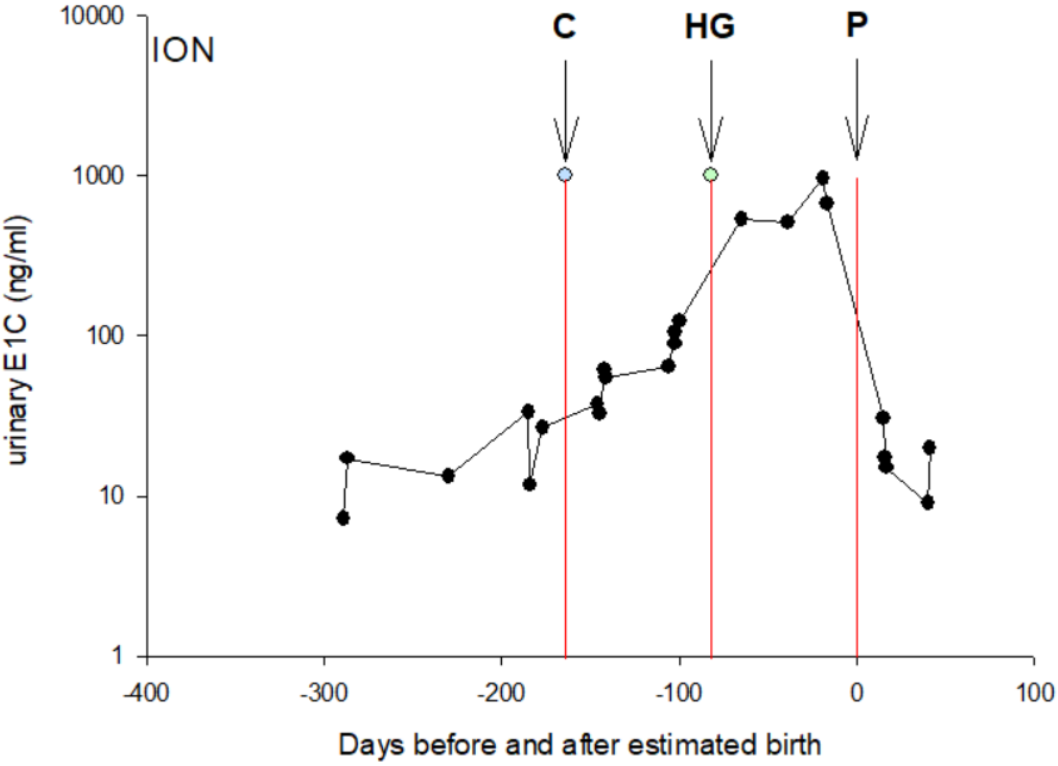

ION
C
HG
P
10000
1000
100
10
1
urinary E1C (ng/ml)
-400
-300
-200
-100
0
100
Days before and after estimated birth

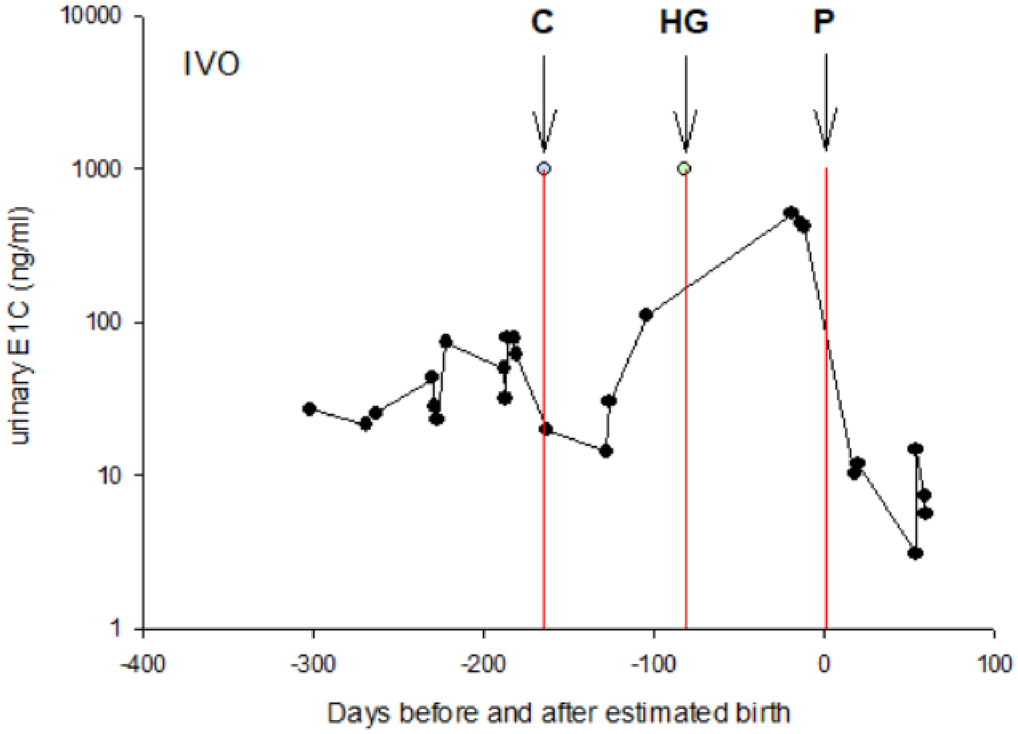
IVO
C
HG
P
urinary E1C (ng/ml)
10000
1000
100
10
1
-400
-300
-200
-100
0
100
Days before and after estimated birth

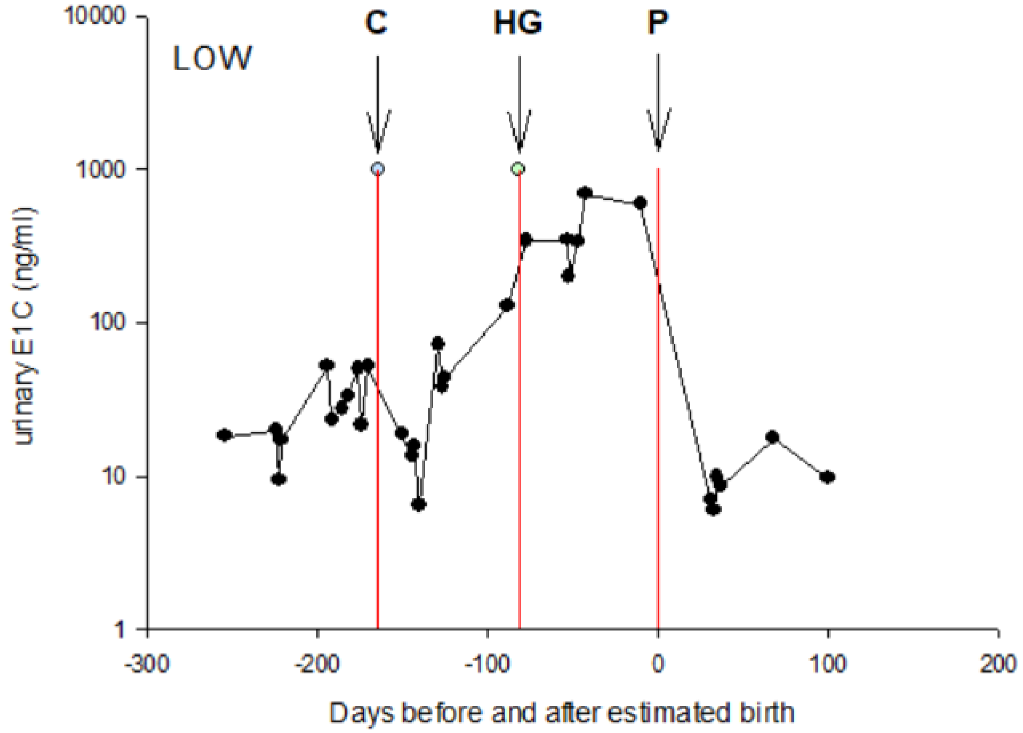
LOW
C
HG
P
urinary E1C (ng/ml)
10000
1000
100
10
1
-300
-200
-100
0
100
200
Days before and after estimated birth

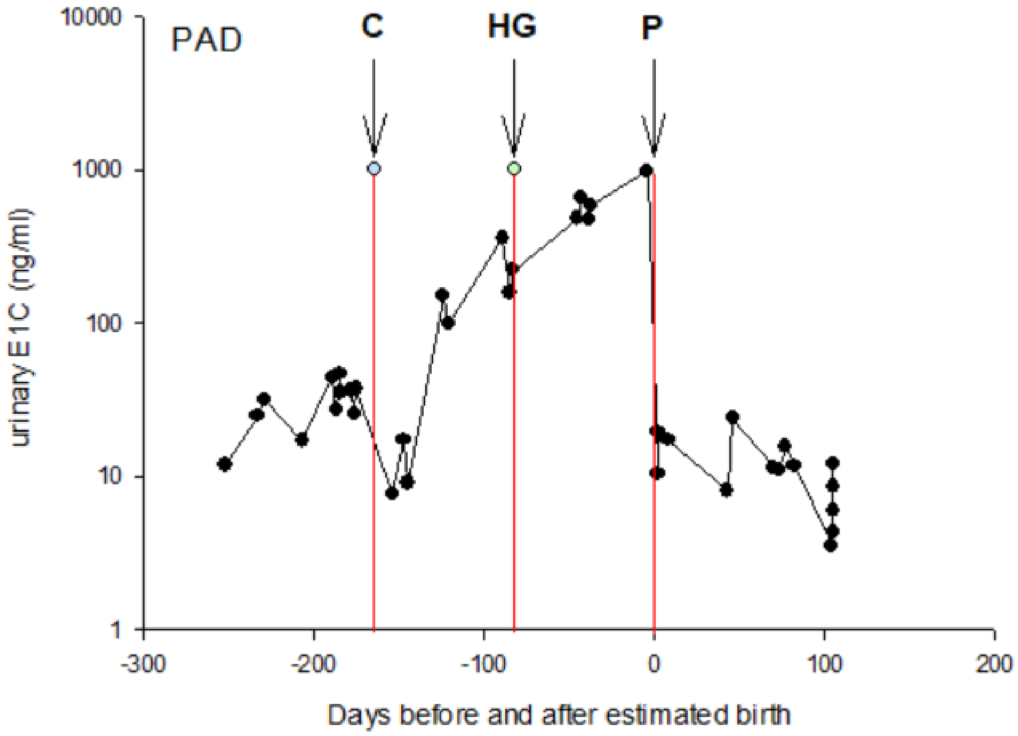

PAD
C
HG
P
10000
1000
100
10
1
urinary E1C (ng/ml)
-300
-200
-100
0
100
200
Days before and after estimated birth

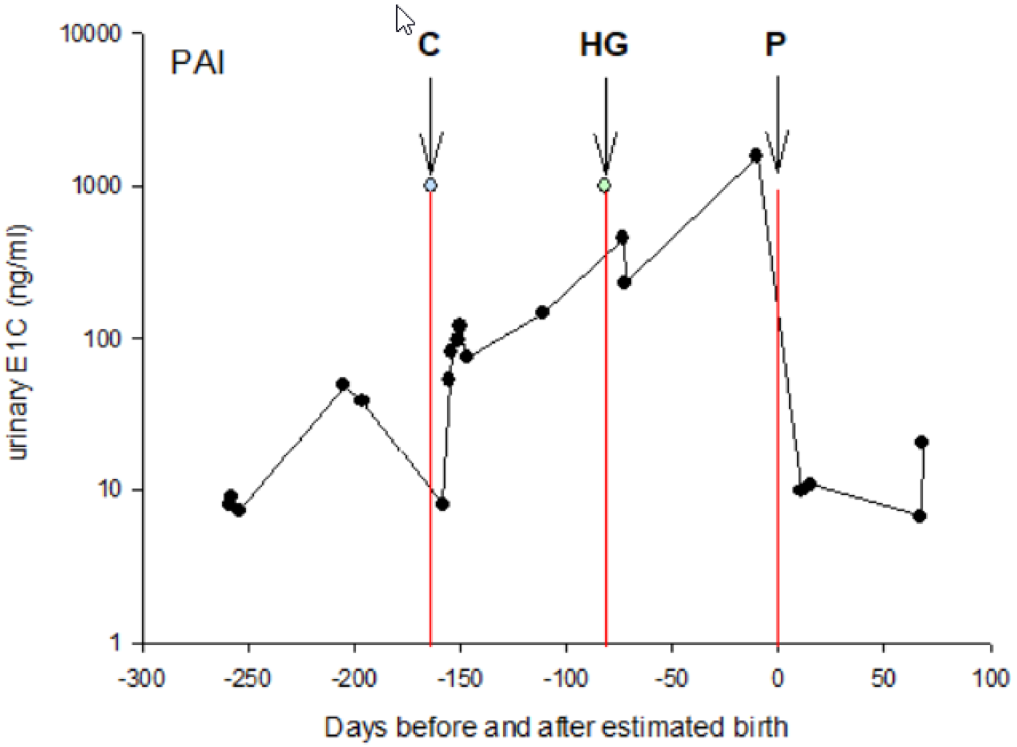

PAI
C
HG
P
10000
1000
100
10
1
urinary E1C (ng/ml)
-300
-250
-200
-150
-100
-50
0
50
100
Days before and after estimated birth

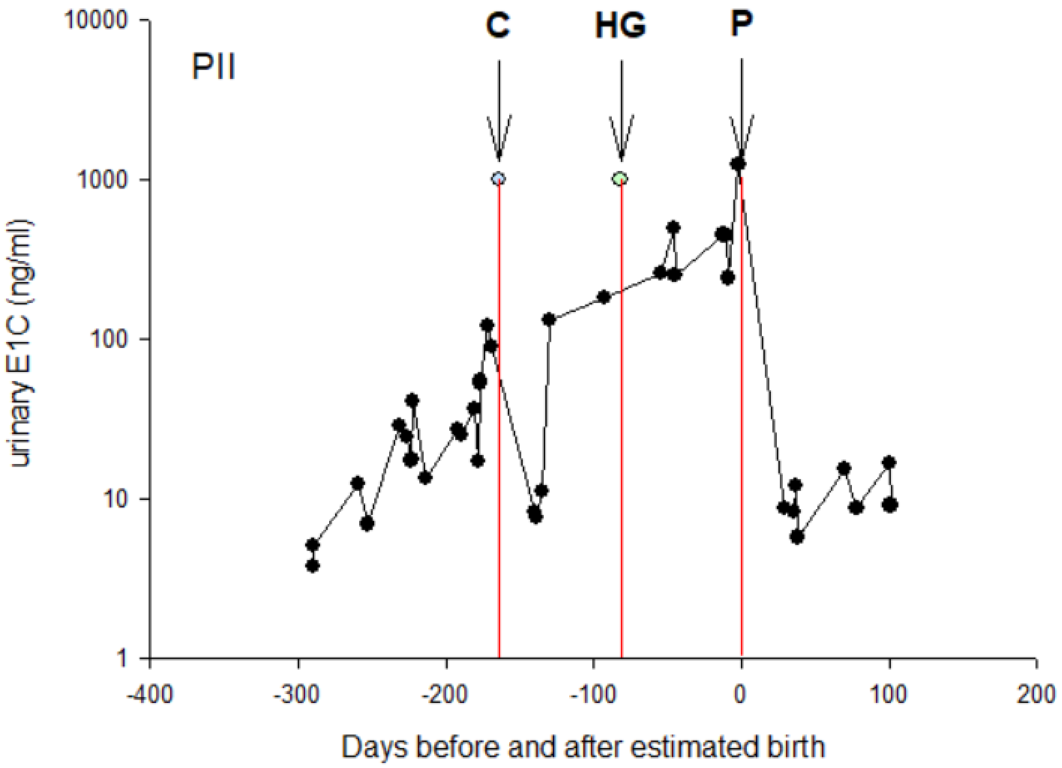
PII
C
HG
P
urinary E1C (ng/ml)
10000
1000
100
10
1
-400
-300
-200
-100
0
100
200
Days before and after estimated birth

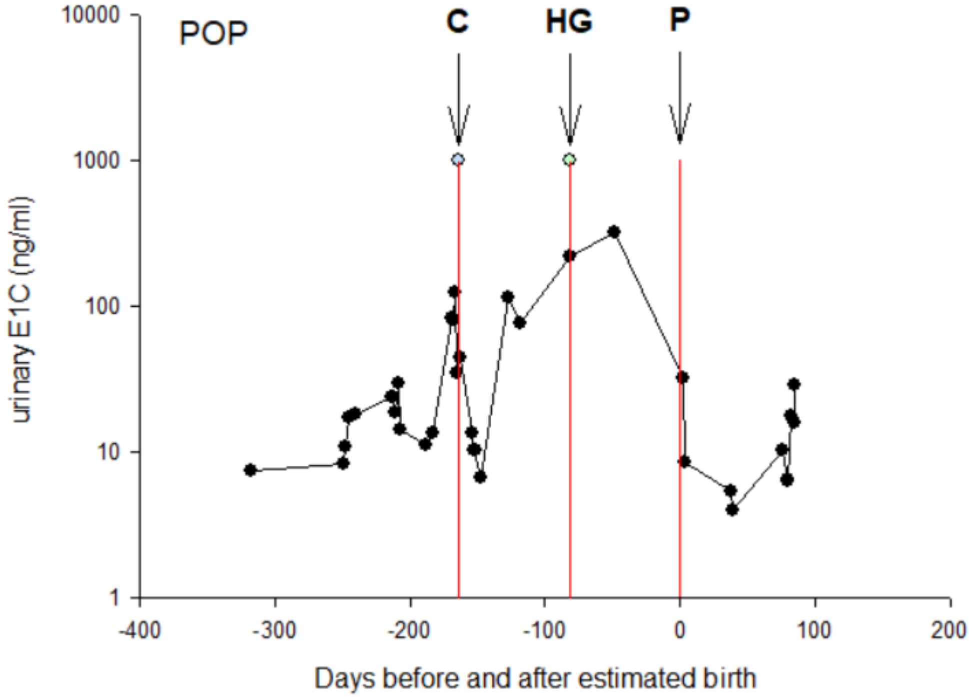
POP
C
HG
P
urinary E1C (ng/ml)
10000
1000
100
10
1
-400
-300
-200
-100
0
100
200
Days before and after estimated birth

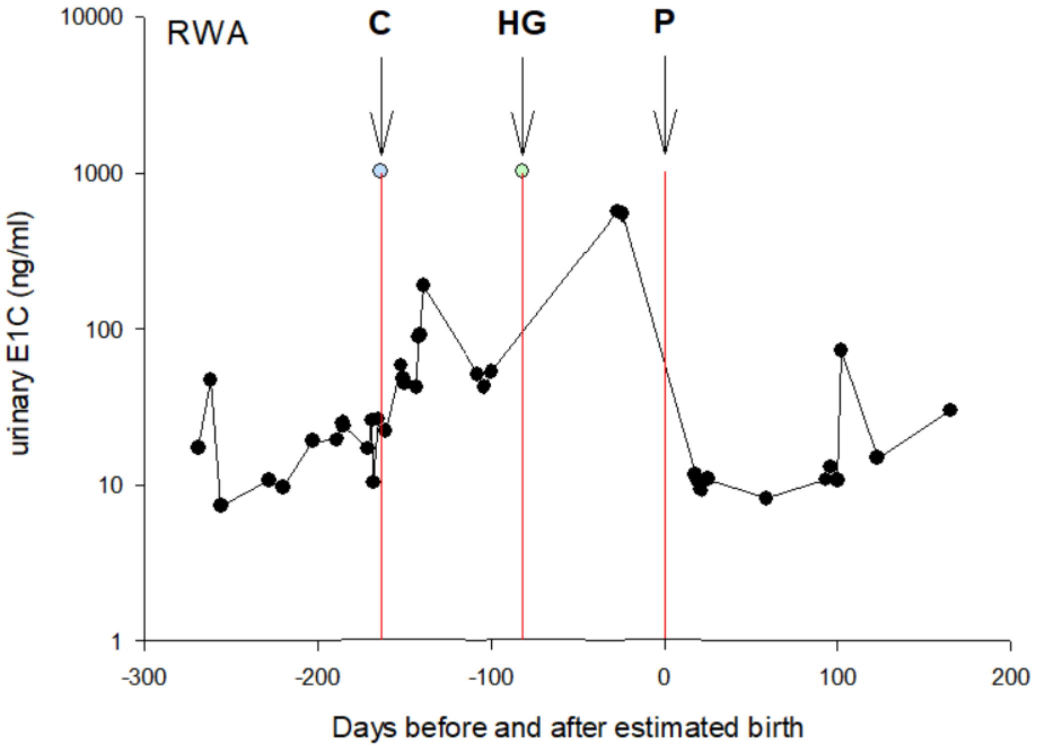

RWA
C
HG
P
urinary E1C (ng/ml)
10000
1000
100
10
1
-300
-200
-100
0
100
200
Days before and after estimated birth

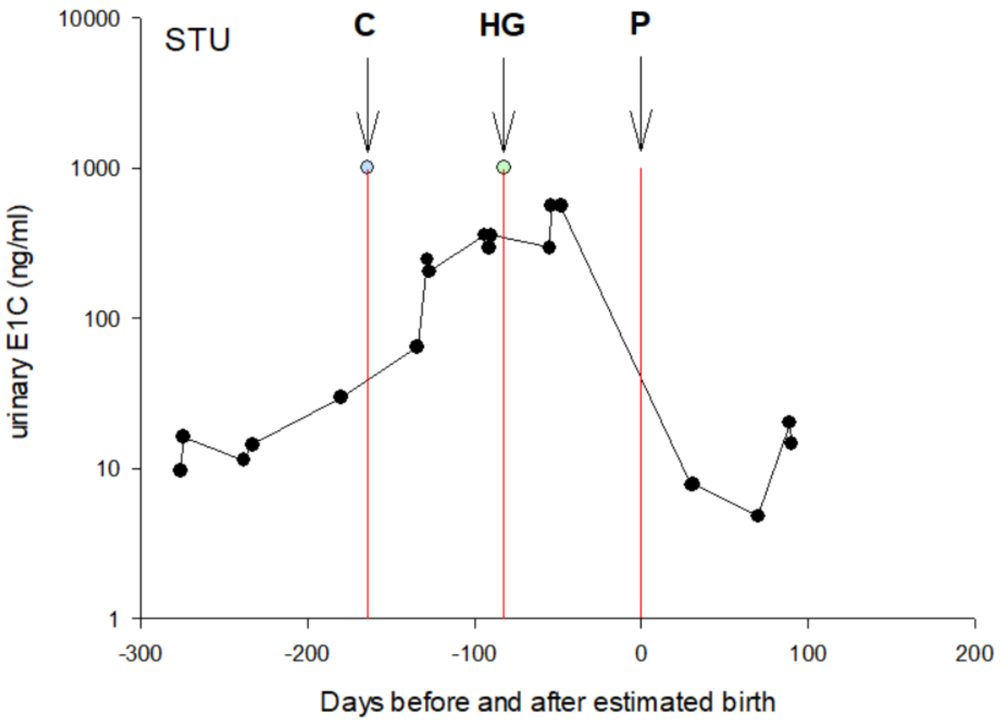

STU
C
HG
P
urinary E1C (ng/ml)
10000
1000
100
10
1
-300
-200
-100
0
100
200
Days before and after estimated birth

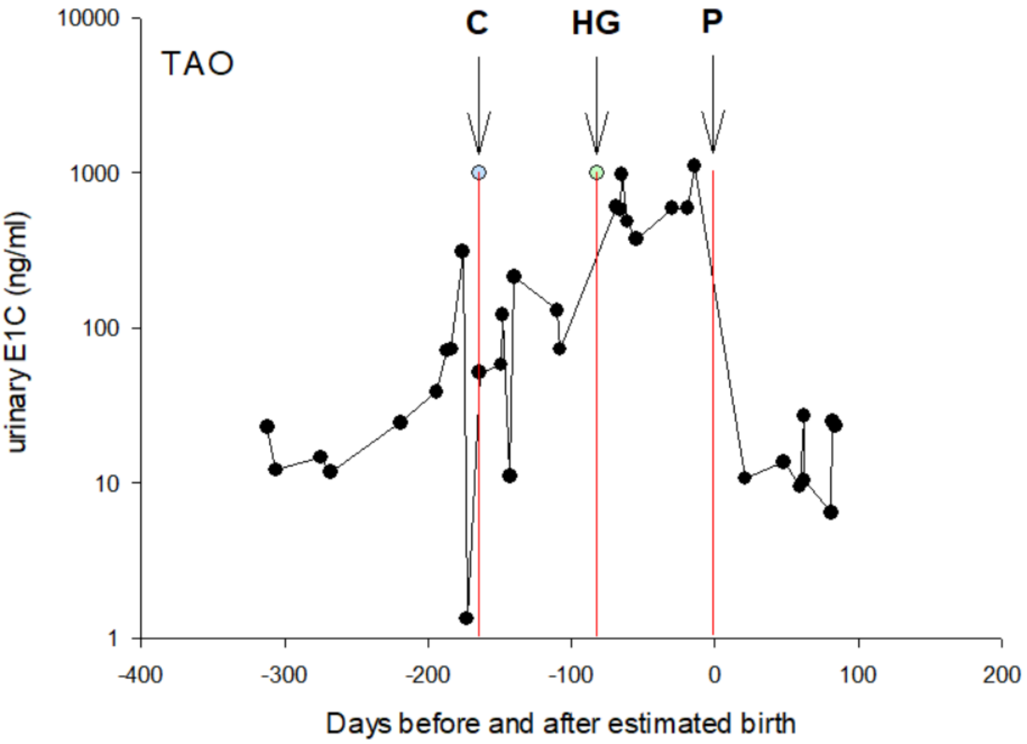
TAO
C
HG
P
10000
1000
100
10
1
urinary E1C (ng/ml)
-400
-300
-200
-100
0
100
200
Days before and after estimated birth

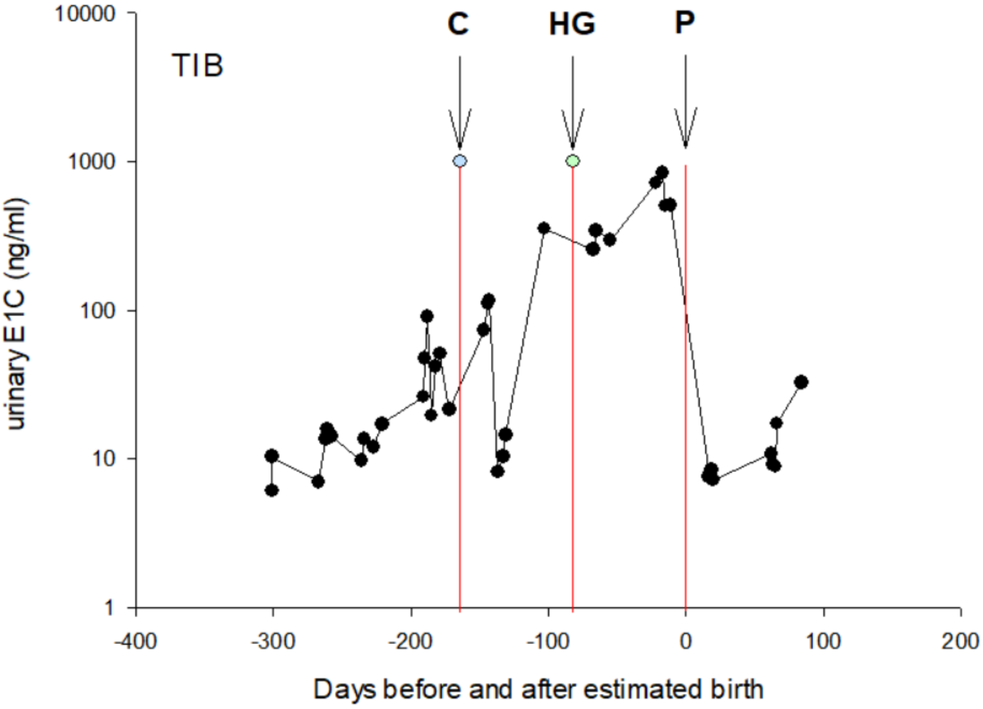
TIB
C
HG
P
10000
1000
100
10
1
urinary E1C (ng/ml)
-400
-300
-200
-100
0
100
200
Days before and after estimated birth

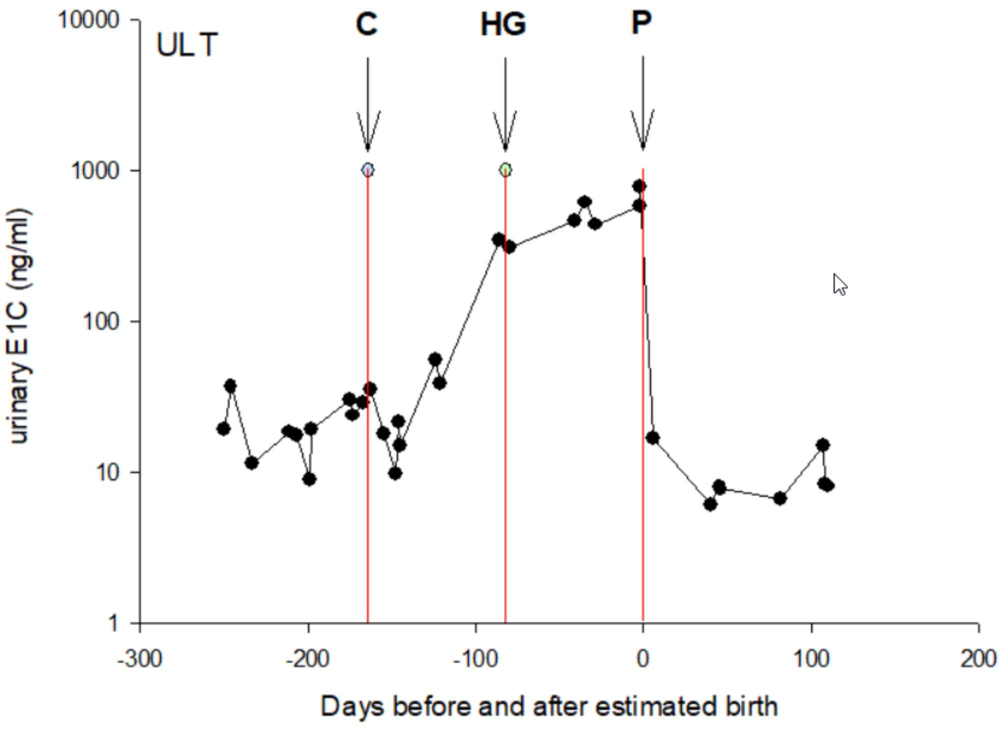

ULT
C
HG
P
urinary E1C (ng/ml)
10000
1000
100
10
1
-300
-200
-100
0
100
200
Days before and after estimated birth

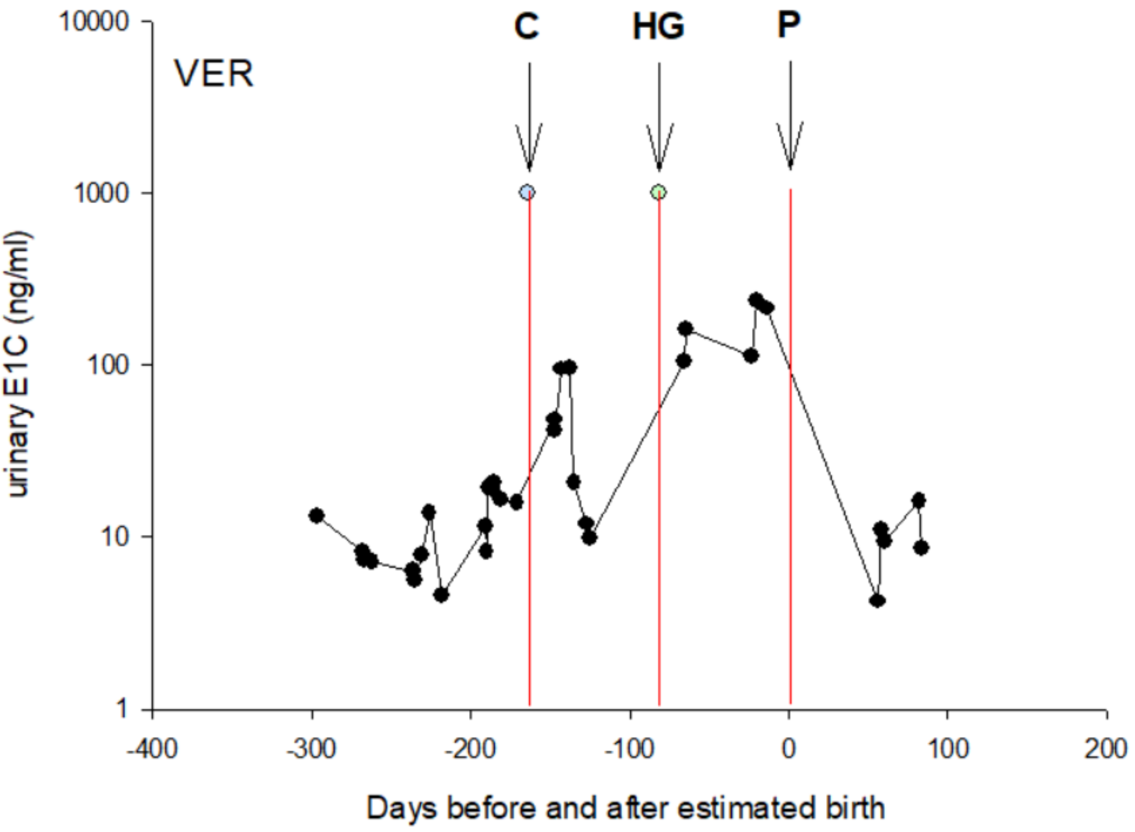

VER
C
HG
P
urinary E1C (ng/ml)
10000
1000
100
10
1
-400
-300
-200
-100
0
100
200
Days before and after estimated birth

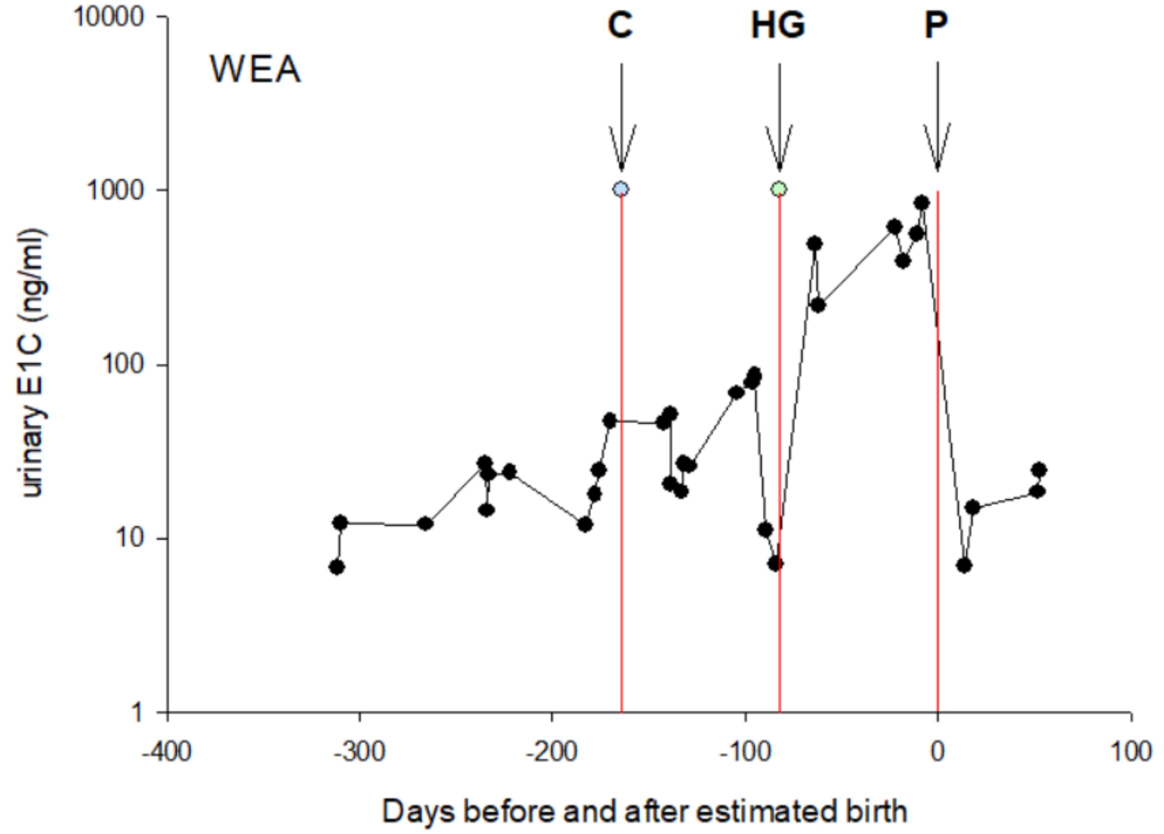

WEA
C
HG
P
urinary E1C (ng/ml)
10000
1000
100
10
1
-400
-300
-200
-100
0
100
Days before and after estimated birth
