# Supplementary material for: Assessing Gestation and Fetal Sex in Wild Assamese Macaques Using Urinary Estrogen Analysis
Source: Am J Primatol. 2025 Aug 20;87(8):e70065. doi: 10.1002/ajp.70065 (PMC12367238; doi:10.1002/ajp.70065)
Supplement: Supplementary file 2 — Supplement Table S1. [file AJP-87-e70065-s001.pdf]

Supplement

Table S1: Distribution of urine sample collection across 19 wild female Assamese macaques displayed per reproductive stage (pre-, early-, late- and post-gestation).

Female	Pre	Early	Late	Post	Sum
**1**	13	1	5	10	29
**2**	7	4	3	12	26
**3**	14	3	3	7	27
**4**	18	10	5	8	41
**5**	11	8	5	14	28
**6**	5	8	4	6	23
**7**	11	1	3	11	26
**8**	13	4	6	8	31
**9**	23	4	6	5	38
**10**	6	6	3	4	19
**11**	18	3	7	10	38
**12**	17	3	2	10	32
**13**	19	10	2	6	37
**14**	5	6	3	5	19
**15**	12	6	8	7	33
**16**	14	4	7	14	39
**17**	16	4	6	7	33
**18**	11	4	5	14	34
**19**	12	6	6	9	33
